# Targeting macrophages in cancer immunotherapy

**DOI:** 10.1038/s41392-021-00506-6

**Published:** 2021-03-26

**Authors:** Zhaojun Duan, Yunping Luo

**Affiliations:** 1 Department of Immunology, Institute of Basic Medical Sciences, Chinese Academy of Medical Sciences; School of Basic Medicine, Peking Union Medical College, Beijing, China; 2 Collaborative Innovation Center for Biotherapy, Institute of Basic Medical Sciences, Chinese Academy of Medical Sciences; School of Basic Medicine, Peking Union Medical College, Beijing, China

**Keywords:** Tumour immunology, Drug development

## Abstract

Immunotherapy is regarded as the most promising treatment for cancers. Various cancer immunotherapies, including adoptive cellular immunotherapy, tumor vaccines, antibodies, immune checkpoint inhibitors, and small-molecule inhibitors, have achieved certain successes. In this review, we summarize the role of macrophages in current immunotherapies and the advantages of targeting macrophages. To better understand and make better use of this type of cell, their development and differentiation characteristics, categories, typical markers, and functions were collated at the beginning of the review. Therapeutic strategies based on or combined with macrophages have the potential to improve the treatment efficacy of cancer therapies.

As a type of phagocytic cell that was initially identified as clearing foreign pathogens by Elie Metchnikoff, macrophages have gradually been considered for cancer immunotherapy in recent years. In light of their positive roles in current therapeutic strategies, they have become a promising target for improved cancer treatments. To facilitate the use of macrophages in cancer immunotherapy, we summarize their related characterization and research progress in this review.

## Categories and characterization of macrophages

### Development and differentiation of macrophages

**Fig. 1:**
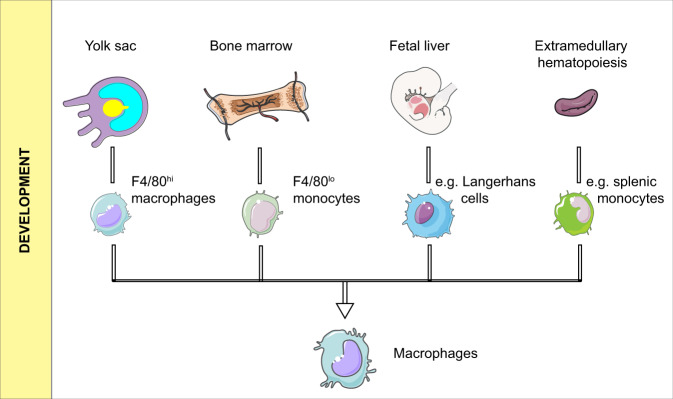
Development of macrophages

It is now widely accepted that macrophages in tissues, as well as monocytes in the peripheral blood, are classified as the mononuclear phagocytic system (MPS). This concept has developed over a long history, and its current version takes the origin, morphology, function, and kinetics of the cells into consideration.^1^ In MPS, macrophages originate from bone marrow stem cells, and their development goes through sequential stages as granulocyte–monocyte progenitor cells, pro-monocytes, and mature monocytes. After entering various tissues, monocytes differentiate into macrophages.^2^ However, in some lower multicellular organisms without circulating monocytes, such as Porifera, macrophages still exist. For patients with monocytopenia, their macrophages do not diminish correspondingly.^3^ These phenomena indicate that macrophages could come from other sources in addition to monocytes. This notion has been supported by additional studies. As shown in Fig. 1, based on studies from mouse models, macrophages possibly have at least four origins:^4,5^ (1) F4/80^hi^ macrophages from the yolk sac that mainly reside in tissues such as the liver, spleen, lung, brain, pancreas, and kidney; (2) F4/80^lo^ macrophages derived from bone marrow and developed through a mature stage as Ly6C^+^ monocytes; (3) Langerhans cells from the fetal liver (regarding Langerhans cells as macrophages but not DC cells^6^); (4) A few studies also have claimed that a minority of tumor-associated macrophages may come from extramedullary hematopoiesis, especially in the spleen.^7,8^ It has been reported that Ly6C^−^ patrolling monocytes are mainly responsible for detecting pathogens intravascularly and maintaining vascular integrity, while Ly6C^+^ inflammatory monocytes are recruited to infectious sites and injuries, mediating extravascular inflammatory responses and then differentiating into macrophages.^4,9^ Some studies have also indicated that both Gr1^+^/Ly6C^high^^10–12^ and Gr1^−^/Ly6C^lo^ monocytes have the potential to enter tissues and turn into macrophages,^13^ but the former are more likely to become M1 macrophages, while the latter are M2 phenotypes.^14^ Above all, macrophages in tissues are probably a mixture of embryo- and adult-derived cells.

Wherever the macrophages originated from, the macrophage colony-stimulating factor 1 receptor (CSF1R) is a key receptor that induces their differentiation. CSF1 and IL-34 are two ligands of CSF1R. These two factors function in different ways. It has been reported that macrophages in the liver, spleen, or bone marrow are typically regulated by CSF1-mediated signals,^15^ while IL-34 dominates the development of macrophages in the brain.^16^ Given the importance of CSF1R, its inhibitors are often used in scientific studies to deplete macrophages. In addition, the lack of Sfpi1, which is a pioneering transcriptional regulator in myeloid lineage development, could result in a total depletion of CD11b^+^F4/80^+^ macrophages.^17^ An expression disparity of Sfpi1 determines the differentiation of Ly6C^hi^ monocytes into iNOS^+^ macrophages or monocyte-derived dendritic cells.^18^ Id3 is indispensable for liver macrophages.^19^ PPARγ maintains the anti-inflammatory function of alveolar macrophages.^20^ Gata6 controls the proliferative renewal of peritoneal macrophages.^21^ LXR deficiency could cause a failure in the generation of splenic marginal zone macrophages and metallophilic macrophages.^22^ Epigenetic changes drive the differentiation of monocytes into macrophages.^23^ More factors participating in the differentiation of macrophages have been described in previous reviews.^4,24,25^

### Categories

Macrophages are widely distributed in various tissues. According to their histological locations, macrophages residing in specific tissues can be categorized into Kupffer cells in the liver, microglial cells in the brain, osteoclasts in the osseous tissue, alveolar macrophages in the lung, mesangial cells in the kidney, subcapsular sinus macrophages in lymph, and so on.^26,27^ A summary of the ontogeny, functions, and markers of macrophages in different tissues is listed in Table 1. It has been shown that macrophages from different tissues possess diverse expression profiles for transcripts and proteins, which can have a profound impact on their phenotypes and functions.^28,29^

**Table 1:** Ontogeny, functions and identifying markers of different macrophages

Tissue	Macrophage	Ontogeny	Function	Identifying markers	Refs.
Liver	Kupffer cells	Yolk sac derived	Clearance of bacteria, aged erythrocytes, and cellular debris from the blood; regulation of the immune response; involvement in liver injury repair	F4/80^hi^ CD11b^lo^ Siglec-1^+^ CD68^+^ Galectin-3^+^ CD80^lo/−^ PPARδ^+^ Ly6C^−^ CX3CR1^−^ Clec4F^+^ TIM-4^+^	^27,62,223–225^
	Monocyte-derived liver macrophage (MoMFs)	Monocyte derived	Rapid accumulation and involvement in immune responses after organ damage	F4/80^+^ CD11b^hi^ MHC-II^+^ CCR2^lo^ (transferring from CCR2^hi^) CD64^+^ CX3CR1^hi^ (transferring from CX3CR1^lo^)	^226–229^
	Liver capsular macrophages	Monocyte derived	Sensing bacteria reaching the hepatic capsule; inhibition of the hepatic spread of peritoneal pathogens; recruiting neutrophils	F4/80^+^ MHC-II^+^ CD11b^+^ CD64^+^ CD103^−^ CX3CR1^+^ TIM-4^−^ CD207^+^	^230^
Lung	Alveolar macrophages	Yolk sac and fetal liver progenitors	Immune surveillance; phagocytosis of inhaled particles	F4/80^lo^ CD11b^lo^ CD11c^hi^ CD14^lo^ CD200R^hi^ DEC205^inter^ MHC-II^lo^ CD68^+^ Siglec F^+^ MARCO^+^ CD206^+^ Dectin-1^+^ Galectin-3^+^ Mertk^+^ CD64^+^ Siglec-1^+^	^27,223,231–233^
	Interstitial macrophages	Fetal liver and bone marrow-derived monocytes	Immune surveillance	F4/80^+^ CD11b^+^ CD11c^+^ CD68^+^ MHC-II^+^ CD24^−^ CD86^+^ Ly6C^−^ Siglec F^-^ CD64^+^	^233–236^
Central nervous system	Microglial cells	Yolk sac derived	Functioning as immune surveillance; promote neuronal survival and remove dead neurons; synaptic remodeling	F4/80^+^ CD11b^+^ CD45^lo^ CX3CR1^hi^ Iba-1^+^ P2RY12^+^	^26,27,236,237^
	Perivascular macrophages	Yolk sac or fetal liver progenitors	Blood–brain barrier integrity; phagocytosis; antigen presentation; lymphatic clearance	CD45^hi^ CD11b^+^ F4/80^hi^ CX3CR1^hi^ Iba-1^hi^ P2RY12^−^ CD163^+^ CD206^+^ Lyve-1^+^	^237–248^
	Meningeal macrophages	Yolk sac derived	Immune surveillance	F4/80^+^ CD11b^+^ CD45^hi^ CX3CR1^hi^ Iba-1^+^ CD209b^+^ Chnrb4^+^	^27,237,249^
Bone	Osteoclast	Monocyte derived	Resorption of organic matter and minerals from the bone matrix	Calcitonin receptor^+^ Calcr^+^ RANKL^+^	^26,27,250–252^
	Bone marrow macrophages	Yolk sac derived or fetal liver-derived monocytes	Promoting erythropoiesis; maintenance of the hematopoietic stem cells niche	Siglec-1^+^ ER-HR3^+^ F4/80^+^ Tartrate-resistant acid phosphatase (TRAP)^−^	^250,253^
Spleen	Marginal zone macrophages	Bone marrow-derived monocytes	Clearance of pathogens present in the circulation; retention of marginal zone B cells	CD68^+^ Dectin-2^+^ F4/80^lo^ LXRα^+^ MARCO^+^ TIM-4^+^ SIGN-R1^+^	^22,254–256^
	Marginal metallophilic macrophages	Bone marrow-derived monocytes	Clearance of pathogens present in the circulation	CD68 ^+^ F4/80^lo^ LXRα^+^ Siglec-1^+^	^257^
	White pulp (tingible body) macrophages	Not clear	Clearance of apoptotic B cells	CD68 ^+^ MFG-E8^+^ Mertk^+^ TIM-4^+^ CD36^+^	^257–259^
	Red pulp macrophages	Yolk sac-derived or fetal liver-derived monocytes	Clearance of effete red blood cells; immunosurveillance; detoxification; iron recycling; antigen delivery to DCs	F4/80^hi^ CD11b^lo^ Siglec-1^lo^ CD68^+^ MHC-II^lo^ CSF1R^+^ SIRPα^+^ Siglec F^−^ CD163^+^ Dectin-2^+^ VCAM1^+^ Spi-C^+^ Heme Oxigenase^+^ Ferroportin^+^	^223,255,259–261^
Kidney	Mesangial cell	Monocyte derived	Intraglomerular mesangial cells; regulation of glomerular filtration; mesangial matrix formation; phagocytosis; monitoring of glucose concentrations	F4/80^+^ CD11b^lo^ CD103^−^ CX3CR1^+^ SIRPα^+^ Siglec F^-^	^223^
Lymph node	Subcapsular sinus macrophages	Yolk sac-derived or bone marrow-derived monocyte	Limiting the systemic dissemination of pathogens and bacterial infections; promote the presentation of antigens	F4/80^lo^ MARCO^+^ Siglec-1^hi^ CD11b^hi^ Ligands for the cysteine-rich domain of the mannose receptor^+^	^223,262,263^
	Medullary macrophages	Bone marrow-derived monocytes	Highly phagocytic and rapidly clear pathogens	CD11b^+^ Siglec-1^+^ F4/80^+^ MARCO^+^ SIGN-R1^+^	^223,263,264^
Serosal Tissues	Pleural macrophages	Bone marrow-derived monocytes	Immune surveillance	CD11b^hi^ F4/80^hi^ Siglec F^−^ RELMα^+^ TIM-4^+^	^265–267^
	Large peritoneal macrophages	Yolk sac-derived or fetal liver-derived monocytes	Regulation of IgA production in the gut by peritoneal B1 cells	CD11b^hi^ CD11c^lo^ SIGN-R1^−^ F4/80^hi^ GATA-6^+^ MHC-II^lo/−^ CD62L^–^ TIM-4^+^	^268–270^
	Small peritoneal macrophages	Bone marrow-derived monocytes	Immune surveillance	CD11b^lo^ CD11C^−^ SIGN-R1^+^ F4/80^lo^ MHC-II^hi^ CD62L^+^ TIM-4^-^	^265,269,270^
Skin	Langerhans cells	Yolk sac-derived or fetal liver-derived monocytes	Interaction with T lymphocytes; immune surveillance	CD11b ^+^ CD11c^+^ F4/80^+^ Id2^+^ Langerin^+^ RUNX3 ^+^	^27,271,272^
	Dermal macrophages	Bone marrow-derived monocytes	Immune surveillance	CD11b^+^ CD11c^lo^ CD301^+^ Dectin-1^+^ Dectin-2^+^ F4/80^+^ CD64^hi^ Mertk^+^ MHC-II^lo^ CD206^+^ Siglec-1^hi^	^27,223,255,273^
Adipose Tissue	Adipose tissue-associated macrophages	Not clear	Adipogenesis; adaptive thermogenesis; regulation of insulin sensitivity and glucose tolerance	CD45^+^ F4/80^+^ PPARγ^+^	^274,275^
Gastrointestinal Tract	Intestinal lamina propria macrophages	Bone marrow-derived monocytes	Maintenance of intestinal homeostasis; recognition and removal of intestinal pathogens; maintenance of gut epithelial integrity	CD11b^+^ CD11c^+^ CX3CR1^hi^ F4/80^+^ CD64^+^ MHC-II^hi^	^27,276^
Blood	Ly6C^lo^ monocytes	Bone marrow-derived monocytes	Immune surveillance; maintenance of vascular integrity	CD11b^hi^ CD43^+^ CX3CR1^+^ F4/80^+^ Ly6C^lo^ CSF1R^+^ NR4A1^+^	^27,277,278^
Tumor	Tumor-associated macrophage	Yolk sac derived or monocyte derived	Promote tumor growth; inhibit tumoricidal immune response; initiate angiogenesis; activate matrix remodeling; aid invasion and intravasation	Murine: Ly6C^+^ MHC-II^+^ CX3CR1^+^ CCR2^+^ CD62L^+^ TIE2^+^ Human: CD14^+^ CD312^+^ CSF1R^+^ CD16^+^	^42,279–281^

**Fig. 2:**
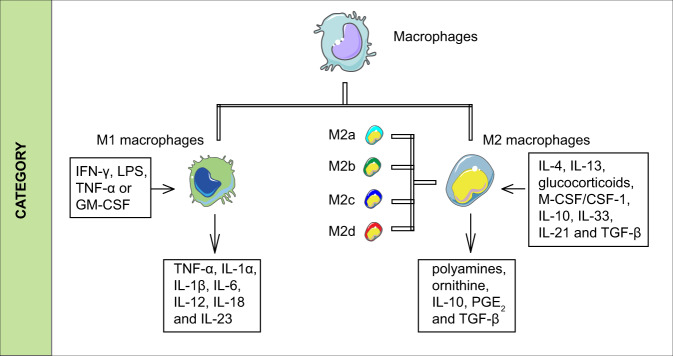
Categories of macrophages

Based on phenotypes and functions, macrophages can be typically divided into M1 (proinflammatory, classically activated macrophages) and M2 (anti-inflammatory, alternatively activated macrophages) types (Fig. 2). In brief, M1 macrophages can be induced by IFN-γ, lipopolysaccharide (LPS), TNF-α or granulocyte–macrophage colony-stimulating factor (GM-CSF), followed by activation of Toll-like receptor signaling pathways. They play a positive role in the removal of pathogens and tumor cells. On the one hand, M1 macrophages express high levels of antigen-presenting MHC complexes, which accelerate the activation of adaptive immune responses. On the other hand, they act directly on target cells by generating nitric oxide, reactive oxygen species, and reactive nitrogen species. Moreover, M1 macrophages promote inflammatory responses by secreting cytokines such as TNF-α, IL-1α, IL-1β, IL-6, IL-12, IL-18, and IL-23.^30,31^ Excessive M1 macrophage-mediated responses may lead to tissue damage, which is the main cause of atherosclerosis and other chronic inflammation.^32–34^ M2 macrophages can be induced by cytokines, such as IL-4, IL-13, glucocorticoids, M-CSF/CSF1, IL-10, IL-33, IL-21, and TGF-β.^31,35–37^ Accompanied by increased production of polyamines and ornithine through the arginase pathway, high secretion of IL-10, PGE_2_, TGF-β, but low IL-12, they are major participants in the clearance of parasites and homeostasis, such as tissue remodeling and regeneration, wound healing and anti-inflammation.^38,39^ When M2 macrophages develop further, they are refined into M2a, M2b, M2c, and M2d subgroups.^40^ Their specific characterizations have been reviewed by Abbas Shapouri Moghaddam et al.^41^ Macrophages have strong plasticity. It has been shown that different phenotypes could possibly transform mutually under certain conditions.

Tumor-associated macrophages (TAMs) generally represent a major component of myeloid cells present in tumors. For some solid tumors, TAMs can arise from several origins: as residual macrophages derived from the yolk sac, infiltrating macrophages as the major replenishment recruited from bone marrow/Ly6C^+^-circulating monocytes, and a minority from the spleen.^8,42–47^ It has been demonstrated that TAMs with different origins may act differently than anti-macrophage oncotherapies.^43^ In most established tumors, TAMs tend to be considered M2-skewed macrophages because they possess the majority of the representative properties of M2 macrophages, usually including but not limited to high expression levels of arginase-1, mannose receptor, and a low MHC class II complex.^48^ Transcriptome profile analysis revealed that TAMs are more similar to fetal macrophages but not inflammatory macrophages.^41^ However, as macrophages are plastic, there is also evidence suggesting that TAMs actually have both M1 and M2 expression patterns or expression patterns distinct from those of M1 and M2 macrophages.^49^ Since 90–95% of neoplasms are closely associated with a chronic inflammatory status, it has been suggested that M1 macrophages may induce tumor initiation by creating a mutagenic microenvironment, while M2 macrophages promote malignancy progression.^36,50^ It is also believed that TAMs may exert both tumor-promoting and tumor-inhibiting functions,^51,52^ which make TAMs a potential target for cancer therapies.

### Typical markers

To be distinguished from other immunocytes, macrophages can be characterized by phagocytosis and the expression of CD11b, F4/80, and CSF1R in mice or CD79, CD163, CD16, CD312, and CD115 in humans.^41^ Specifically, to present antigens and activate adaptive immune responses, M1 macrophages often express high levels of MHC class II molecules and costimulatory molecules, such as CD40, CD80, and CD86, while M2 macrophages contain upregulated levels of endocytosis-related receptors, such as the human scavenger receptors CD163 and Stabilin-1 and C-type lectin receptors, including CD206, CD301, detin-1 and CD209.^31^ In addition to the proinflammatory or anti-inflammatory cytokines mentioned above, polarized macrophages generate different types of chemokines. CXCL9, CXCL10, CXCL11, and CCL5 are usually secreted by M1 macrophages to recruit Th1, Th17, and cytotoxic T cells, while CCL2, CCL17, CCL18, CCL22, and CCL24 are produced by M2 macrophages in most cases.^31,38,40^

### Basic functions of macrophages

One of the basic functions of macrophages is phagocytosis. Through phagocytosis, macrophages can clear erythrocytes, apoptotic cells, and effete cells to maintain homeostasis. Neutropenia and splenomegaly may occur when neutrophils and erythrocytes in the spleen and liver cannot be phagocytized.^53^ This type of clearance process is independent of immune responses and is regarded as the fundamental function of macrophages.^54^ When pathogens or aberrant cells, such as tumor cells, are recognized by macrophages, they can be phagocytized and processed into antigen peptides. Macrophages present these peptides to MHC class II molecules on their surface and stimulate T-cell proliferation and activation with the synergistic effect of costimulatory molecules.^55,56^ It has been reported that adult macrophages are primarily responsible for host defense, while fetal macrophages are involved in tissue remodeling.^40^ Macrophages play an important role in the development and homeostasis. For example, microglia are required in almost every precise developmental stage of the central nervous system.^57^ Cardiac macrophages help maintain homeostasis in the steady-state heart by facilitating myocardial conduction.^58^ CCR2^−^ macrophages are instrumental in cardiac recovery, coronary development, and postnatal coronary growth.^59,60^ Impaired activation or depletion of Kupffer cells leads to hepatic steatosis and insulin resistance.^61–63^ Defects in perivascular macrophages can give rise to the unsuccessful establishment of the blood–brain barrier.^64^ It is well known that macrophages are related to many diseases. Here, we will focus on its role in tumors in the following sections.

## Functions of macrophages in cancers

**Fig. 3:**
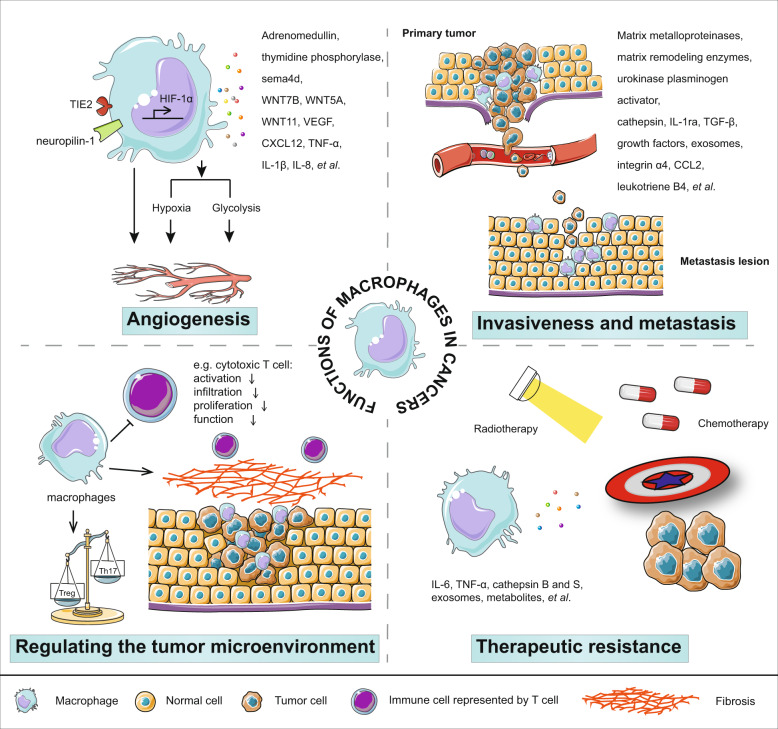
Functions of macrophages in cancers. (1) promotion of angiogenesis; (2) induction of invasiveness and metastasis; (3) regulation of the tumor microenvironment; and (4) induction of therapeutic resistance

By secreting various factors and affecting other immune cells, macrophages not only play a role in chronic inflammation but also initiate, promote, or suppress the development of cancer. Ornithine, VEGF, EGF, and TGF-β are examples of tumor-promoting factors derived from macrophages, while nitric oxide generated by inducible nitric oxide synthase in macrophages can inhibit tumor growth.^32,33,65,66^ Macrophages have been demonstrated to be involved directly or indirectly in several key features of malignant tumors, including angiogenesis, invasiveness, metastasis, regulation of the tumor microenvironment, and therapeutic resistance (Fig. 3).

### Angiogenesis

By expressing WNT7B, WNT5A, WNT11, VEGF-C, VEGF-D, and other factors, macrophages are deeply involved in vasculogenesis and lymphogenesis.^67–70^ In addition, TAMs can enhance tumor hypoxia and glycolysis,^71^ two important causes of angiogenesis.^72,73^ HIF-1a is a protein induced in hypoxia conditions. It has been demonstrated that HIF-1a is an important transcriptional factor regulating the transcription of angiogenesis-associated genes, such as VEGF, bFGF, PDGF, and PGE_2_ in TAMs.^74,75^ Through the synthesis of WNT7B, macrophages also stimulate vascular endothelial cells to produce VEGF.^76^ Other TAM-produced proangiogenic molecules that recruit or activate endothelial cells include CXCL12, TNF-α, IL-1β, IL-8, Sema4d, adrenomedullin, and thymidine phosphorylase.^41,77,78^ Studies on liver diseases have revealed that in addition to producing proangiogenic molecules, macrophages can benefit the formation of complex vascular networks by interacting with the sprouting vasculature.^79^ Live imaging showed that macrophages drive sprouting angiogenesis via VEGF signaling and coordinate blood vessel regression in wound healing by clearing apoptotic endothelial cells.^80^ Preventing macrophages from entering avascular areas by blocking the Sema3A/Nrp1 signaling pathway could inhibit angiogenesis.^81^ It has been reported that angiogenic macrophages are similar to fetal counterparts based on their characteristic expression of TIE2.^77,82^ Targeting TIE2 or its ligand ANG2 inhibits angiogenesis in certain tumor models, such as those for breast and pancreatic cancers.^82^ Depletion of TAMs inhibits angiogenesis.^74,83^ A close relationship between macrophages and angiogenesis has been discussed in previous reviews.^84,85^

### Invasiveness and metastasis

Macrophages can not only increase the density of blood vessels but also promote the invasiveness and metastasis of tumor cells. By expressing matrix metalloproteinases, cathepsin, urokinase plasminogen activator, and matrix remodeling enzymes, such as lysyl oxidase and osteonectin, macrophages dissolve the extracellular matrix to pave the path for tumor cell escape.^4^ TAMs upregulate cytokines, such as IL-1ra, to promote metastasis by enhancing tumor cell stemness.^86^ Secretion of TGF-β and growth factors, such as EGF analogs, promotes epithelial–mesenchymal transition and invasiveness of tumor cells.^87–90^ Exosomes released from M2 macrophages are responsible for cancer metastasis by transferring certain miRNAs into cancer cells, such as colorectal cancer and pancreatic ductal adenocarcinoma cells.^91,92^

In addition to macrophages in primary tumors, macrophages can also assist in tumor survival and colonization at premetastatic lesions. It has been demonstrated that macrophages are required for the early dissemination of breast cancer, and early disseminated macrophages contribute to late metastasis.^93^ Tumor exosomes are crucial in tumor organotropic metastasis. It has been observed that pancreatic cancer cell-derived exosomes preferentially colocalize with macrophages in liver metastasis sites.^94^ Exosome-educated macrophages facilitate premetastatic niche formation via secretion of TGF-β.^95^ In addition, the interplay between integrin a4 on macrophages and VECAM1 on tumor cells promotes cancer cell survival.^96^ Results from other studies support the indispensable role of monocytes/macrophages recruited to premetastatic niches in promoting circulating tumor cell survival and colonization in metastatic lesions.^97,98^ At lung metastasis nodules of breast cancer, CCL2 produced by tumor cells is an important chemokine for the recruitment and retention of inflammatory monocytes/macrophages.^99^ By recruiting Ly6C^+^ monocytes via CCL2, fibrocytes prepare a premetastatic niche in the lung for melanoma cells.^100^ After differentiating of CCR2^+^Ly6C^+^ inflammatory monocytes into Ly6C^−^ macrophages, these cells accelerate tumor cell extravasation by generating VEGF.^101^

Tissue-resident macrophages have also been demonstrated to promote or restrict metastasis. Alveolar macrophages promote hepatocellular carcinoma lung metastasis by producing an inflammatory mediator, leukotriene B4.^102^ By suppressing Th1 responses, alveolar macrophages facilitate breast tumor cells to metastasize.^103^ Kupffer cells engulf cancer cells in a Dectin-2-dependent manner to limit liver metastasis.^104^

### Effects of macrophages on tumor microenvironment

Many factors, such as CSF1, VEGF-A, CXCL12, ANG2, CCL5, and CCL2, in solid tumors, can recruit angiogenic macrophages.^77,101,105–108^ This enrichment allows macrophages to play a major role in the construction of the tumor immune microenvironment. Granulin generated by TAMs can induce fibrosis, which prevents T cells from infiltrating.^109,110^ Attenuation of the TAM antigen presentation ability results in a reduction in T-cell activation and proliferation.^40^ Exosomes consisting of various miRNAs derived from TAMs orchestrate an immunosuppressive tumor microenvironment by causing Treg/Th17 imbalance.^111^ It has been summarized that tumor-associated macrophages support a suppressive tumor microenvironment in three ways:^112^ (1) by consuming the metabolites, (e.g., L-arginine, which is essential for T-cell activation, can be metabolized by TAMs with high expression of ARG1.) (2) by producing the cytokines and chemokines, IL-10, TGF-β, and PGE_2_, which are primarily secreted by TAMs, to inhibit the activation and function of various immune cells, including cytotoxic T cells, but induce and maintain regulatory T cells, (3) by expressing inhibitory molecules. TAMs elicit immune suppression through the expression of inhibitory receptors or immune checkpoint ligands (e.g., MHC-I molecules, PD-L1, PD-L2, CD80, CD86, B7-H4 and VISTA). These molecules deliver an inhibitory signal to ligand- or receptor-expressing immune cells.

### Therapeutic resistance

Macrophages are also an important cell-extrinsic factor that mediates the resistance of tumor cells to chemotherapy or radiotherapy. By expressing IL-6, TNF-α, cathepsin B and S, or inducing other cells to produce IL-6, macrophages activate STAT3 in tumor cells, which enhances the proliferation and survival of malignant cells under treatment with several chemotherapeutics.^113^ The epithelial to mesenchymal transition, which can be elicited by macrophages, has been demonstrated to be another mechanism behind chemoresistance.^114–116^ Exosomal miR-223 from macrophages has been reported to cause a chemoresistant phenotype after being delivered into epithelial ovarian cancer cells.^117^ miR-21 derived from macrophages is responsible for cisplatin resistance in gastric cancer cells.^118^ Macrophages exacerbate fatty acid beta-oxidation of gastric cancer cells by generating growth differentiation factor 15 so that the cancer cells are more resistant to 5-fluorouracil treatment.^119^ Metabolites, including deoxycytidine, from macrophages, weakened the therapeutic effect of gemcitabine in pancreatic ductal adenocarcinoma.^120^ Murine pancreatic ductal adenocarcinoma models showed an enhanced therapeutic response toward gemcitabine after depleting macrophages with liposomal clodronate.^121^ As summarized by Marek Nowak et al., TAMs contribute to chemoresistance by inducing prosurvival and antiapoptotic signals in cancer cells, as well as their protumoral polarization.^122^

It has been reported that irradiation promotes the accumulation and M2 polarization of macrophages.^123^ Heparin-binding epidermal growth factor, which is primarily secreted by macrophages, could reduce the radiosensitivity of head and neck cancer cells by activating the nonhomologous end-joining pathway.^124^ TNF-α has a radioprotective function in a TAM-produced VEGF-dependent manner.^125^ Carcinoembryonic antigen has been identified as a radioresistance marker in colorectal cancer because it induces M2 polarization of macrophages.^126^ Inhibition of differentiation of M2 macrophages showed enhanced responses to radiotherapy in breast cancer.^127^ Of note, dying cancer cells after treatment with chemotherapeutics or radiation might also initiate antitumor immune responses. Whether the function of macrophages leads to sensitization or resistance to traditional therapy is complex.^128,129^ Better understanding of the mechanisms can improve the efficacy of traditional oncotherapy.

## Involvement of macrophages in current immunotherapy

Due to the limitations and shortages of traditional cancer treatments, immunotherapy has become the most promising cancer treatment. Various cancer immunotherapy strategies have emerged. These include adoptive cellular immunotherapy, tumor vaccines, antibodies, immune checkpoint inhibitors, and small-molecule inhibitors. Although most of these strategies are not meant to target macrophages directly or originally, macrophages contribute significantly to the final outcomes.

### Immune checkpoint inhibitors

To date, many immune checkpoint blockade therapies have been reported, but the most commonly used therapies in the clinic are anti-PD-1 and anti-PD-L1 therapies. Cancer immunotherapy based on inhibiting the immune checkpoints CTLA-4 and PD-1 aim at relieving immune suppression rather than simply reinforcing immune responses. Blocking the PD-1/PD-L1 pathways with inhibitors to enhance the cytotoxic function of T cells has made certain achievements in the resolution of malignancies.^130^ However, even if the adaptive immune system is compromised^131^ or the function of T cells cannot be fully recovered by PD-1 inhibitors under specific circumstances,^132^ PD-1/PD-L1 antagonisms can still increase antitumor efficacy. Therefore, more immune cell types should be involved in PD-1/PD-L1 inhibitor treatment. Additional studies revealed that both PD-L1 and PD-1 are expressed in TAMs,^131,133,134^ promoting immune suppression and escape. PD-1^+^ TAM phagocytosis can be rescued by PD-1/PD-L1 blockade, which leads to a direct decrease in tumor burden.^131^ Furthermore, anti-PD-1 or PD-L1 immune checkpoint blockade induced an M1 macrophage polarization.^135,136^ M1 macrophage polarization or repolarization has been linked to an enhanced antineoplastic effect by numerous studies.^137–140^ Of note, macrophages might play a negative role in anti-PD-1 treatment, such as by preventing cytotoxic T cells from reaching tumor cells.^141^ In addition, in vivo imaging showed the transfer of an anti-PD-1 antibody from CD8^+^ T cells to TAMs through the binding of Fc-Fcγ receptors shortly after its administration. Blocking such binding reduced the accumulation of anti-PD-1 antibody in TAMs and prolonged its retention time in CD8^+^ T cells, leading to the regression of tumors.^142^

Along with the concept of immune checkpoints on T cells, several checkpoints that are mainly associated with macrophages have also been discovered. CD47 is a poor prognostic factor in tumor cells, and its interaction with SIRPα on macrophages helps tumor cells evade phagocytic clearance by macrophages.^143,144^ Blocking CD47 has resulted in macrophage-mediated tumor inhibition.^145^ The inhibitory receptor LILRB1 expressed on macrophages prevents tumor cells from being phagocytosed by interacting with the beta-2 microglobulin (β2M) subunit of the MHC class I complex.^146^ The CD24-Siglec-10 axis promotes immune evasion by downregulating macrophage phagocytosis.^147^ Inhibition of these immune checkpoints has significantly increased cancer immunotherapy efficacy.

### Tumor vaccines

Vaccines can be divided into two categories: preventive vaccines and therapeutic vaccines. Preventive vaccines are often designed to induce specific adaptive immunity, chiefly humoral immunity, before the occurrence of disease, which is normally caused by infection with a virus or bacteria. Thus, it can be used to reduce the incidence of viral or bacterial infection-induced carcinoma. Typical examples of preventive vaccines are those for HBV or HPV. Although a proper adaptive immune response is believed to be the primary reason for the effectiveness of these vaccines, it has been reported that immediate innate immunity other than time-consuming adaptive immunity is principally responsible for the spontaneous regression of cancer.^148,149^

Therapeutic vaccines are usually designed to elicit protective T cells. However, Maxime Thoreau et al. demonstrated that cooperation between T cells and macrophages is required to achieve the effects of a therapeutic vaccine. A denser presence of macrophages along with tumor regression has shown to precede the infiltration of CD8^+^ T cells.^150^ Numerous approaches choose synthetic peptides, recombinant proteins, whole tumor cells, viral vectors, bacteria or nucleic acids as vaccination candidates to activate T cells via antigen-presenting cells, which are mostly dendritic cells.^151^ Among these, some regimens that used GM-CSF as an adjuvant generated obvious immune responses.^151,152^ Sipuleucel-T was the first therapeutic vaccine approved by the FDA to be used in a particular group of prostate cancer patients. A fusion protein combining a targeting tumor antigen prostate acid phosphatase with GM-CSF was used to induce antigen-specific T cells. It prolonged the survival of patients in a few clinical trials.^153^ A STING agonist formulated with GM-CSF showed remarkable antitumor efficacy in multiple established tumors.^154^ Some tumor cells used as whole-cell vaccines can secrete GM-CSF.^155,156^ In addition, oncolytic virotherapy, which increases the targeting of cancer cells through virus infection, could induce antitumor immune responses, especially in cells that had been engineered to express GM-CSF.^157,158^ GM-CSF is combined for the purpose of enhancing DC functions and limiting Treg regulation. However, GM-CSF could also induce M1 macrophage polarization and activate macrophages to exert an antitumor function.^40,159,160^ In another virus-related tumor immunotherapy study, Danyang Wang et al. used an NF-κB-activating gene expression adeno-associated virus system to express an artificial neoantigen on the tumor cell surface, which could be targeted by specific immune cells. When they chose calreticulin, a signal to promote phagocytic uptake, the cancer cells could be engulfed by macrophages.^161^ In addition, exosomes derived from M1- but not M2-polarized macrophages boosted the antitumor vaccine by eliciting a release of Th1 cytokines and a stronger antigen-specific cytotoxic T-cell response.^162^ Xu et al. reported that a listeria-based tumor vaccine benefited anti-PD-1 therapy against hepatocellular carcinoma by skewing macrophage polarization.^163^

### Antibodies

Checkpoint inhibitors, such as nivolumab (Opdivo) and pembrolizumab (Keytruda), are monoclonal antibodies. In addition, many other monoclonal antibodies have been approved for clinical cancer immunotherapy by the FDA. Rituximab and trastuzumab are examples of these monoclonal antibodies. Rituximab is used in B-cell lymphoma by targeting CD20. B lymphoma cells are more sensitive to macrophages in the presence of rituximab.^164^ Its combination with cyclophosphamide induced nearly complete tumor elimination in resistant bone marrow by activating macrophages.^165^ After blocking the CD47-SIRPα axis, rituximab-induced macrophage phagocytosis was augmented in nongerminal center B diffuse large B-cell lymphoma patients.^166^ Trastuzumab is an HER2-targeting antibody that has shown promising efficacy in breast cancer therapy. It has been reported that antibody-dependent cell phagocytosis mediated by macrophages is the main cause of the effectiveness of trastuzumab plus CD47 blockade.^167^ By binding with Fcγ receptors on macrophages, trastuzumab triggered macrophage phagocytic killing, and this function was augmented after increasing the expression of Fcγ receptors on macrophages.^168^ In addition, trastuzumab resistance was overcome by shifting macrophages from the M2 to M1 phenotype.^169^

### Adoptive cell therapy

Adoptive cell therapy is also a very promising therapy that induces tumor regression by transferring specific immune cells to the tumor-bearing host. These cells may come from the host itself or some other donors. They are commonly manipulated to possess better effector functions and proliferate to a sufficient number in vitro before administration.^170^ Typical examples include T cells with engineered chimeric antigen receptors (CAR-Ts) or gene-modified T-cell receptors (TCR-Ts). In 2006, the adoptive transfer of TCR-engineered lymphocytes, which recognize an antigen named MART-1, caused tumor regression in metastatic melanoma patients.^171^ In 2010, administration of CAR-T cells against CD19 efficiently eliminated B cells in a patient with follicular lymphoma.^172^ However, insufficient infiltration into solid tumors is a major limitation for these T-cell-based immunotherapies. Local low-dose irradiation increased T-cell recruitment by inducing M1-phenotype macrophage differentiation.^173^ Cytokine release syndrome is considered to be closely related to the efficacy of adoptive cell therapy, but serious cytokine release syndrome may lead to death. It has been reported that cytokine release syndrome induced by CAR-T-cell transfer is mediated by macrophages.^174^ Inhibiting or neutralizing GM-CSF abolished macrophage-derived cytokines, which released syndrome-related cytokines and enhanced CAR-T cell functions.^175,176^ Therefore, taking the response of macrophages into account may benefit adoptive modified T-cell therapy. Modified macrophages with the chimeric antigen receptor (CARMA) have also been tested by Klichinsky et al. The first generation of chimeric antigen receptors, which combine the scFv of anti-CD19, anti-mesothelin, or anti-HER2 antibodies with a CD3 intracellular domain, has been constructed. This CARMA displayed a strong tumoricidal function in preclinical models.^177^

### Small-molecule inhibitors

Because of several advantages, such as oral bioavailability, the relatively low cost, ease of crossing physiological barriers or access to intracellular targets, small-molecule drugs are complementary and synergistic with other immune-oncology therapies.^178^ Numerous small-molecule inhibitors have been proven to suppress tumors by targeting macrophage-associated molecules. For example, IDO is a poor prognosis indicator that is often highly expressed in macrophages, dendritic cells, and tumor cells. Small-molecule inhibitors targeting IDO have been tested in clinical trials to reestablish positive immune responses.^179,180^ ARG1 is a cytosolic enzyme that plays a key role in the immunosuppressive function of TAMs. Compounds inhibiting arginase have shown potential in tumor suppression.^181^ RRX-001, a small-molecule inhibitor, downregulated not only CD47 on cancer cells but also SIRPα on macrophages and showed hypotoxicity but strong antitumor activity in clinical trials.^182^ In addition, small-molecule inhibitors have great potential in combination with other oncotherapy strategies. Inhibition of Bcl-2 family members improved the efficacy of CAR-T therapy in B-cell malignancy.^183^ PI3K-γ inhibitors, such as IPI-549, overcome immune checkpoint resistance by reshaping the tumor microenvironment, including switching macrophage polarization from the M2 to M1 phenotype.^184^ Small-molecule inhibitors targeting CXCR2 on neutrophils and CCR2 on macrophages improve the chemotherapeutic effects in pancreatic ductal adenocarcinoma models.^185^ PLX-3397, a small-molecule inhibitor of CSF1R, cKIT, and FLT3 has been demonstrated to decrease tumor burden by reducing M2 macrophages in combination with adoptive cell transfer immunotherapy or other small-molecule inhibitors.^186,187^ FAK is indispensable for the migration and stable protrusion formation of macrophages. Small-molecule inhibitors against FAK have shown promising antitumor activity, especially when combined with chemotherapy and immunotherapy strategies.^188^

## Prospect: macrophages are a promising target in future cancer immunotherapy

**Fig. 4:**
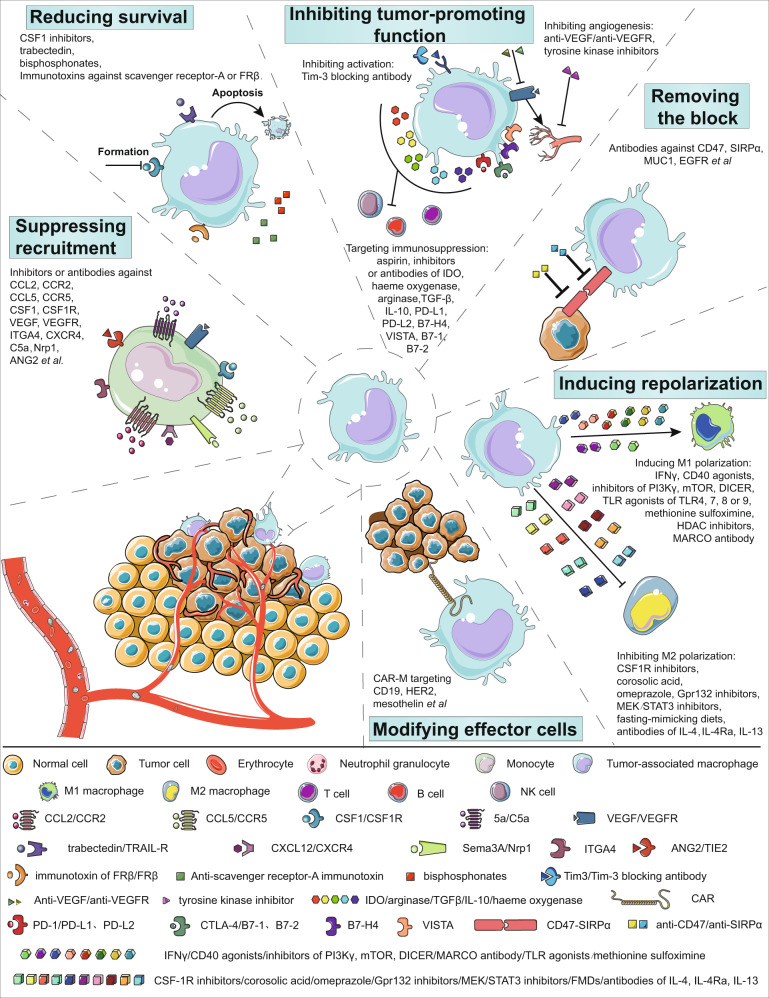
Strategies for targeting macrophages for tumor immunotherapy. These strategies are categorized into six types based on their objectives. Agents or drugs are listed as examples in the subcategory for one of their main effects. This may not be the only effective because of their complex mechanisms. (1) Suppression of macrophage recruitment;^81,82,205^ molecules on monocytes/macrophages, such as CCR2, CCR5, VEGFR, CSF1R, ITGA4, and C5a, contribute to the infiltration of macrophages into tumors. Inhibitors or antibodies against them or some of their ligands (such as CCL2, CCL5, VEGF, and CSF1) could suppress the recruitment of macrophages. Reduced angiogenesis caused by targeting Nrp1 and ANG2 could also result in a decrease in macrophage recruitment. (2) Reduction of macrophage survival.^205–208^ As CSF1 is a crucial signal for the differentiation of macrophages, CSF1 inhibitors restrain the formation of macrophages. Trabectedin could also be used to reduce the survival of macrophages by inducing apoptosis. Immunotoxins targeting scavenger receptor-A or folate receptor β (FRβ) can deplete TAMs, and bisphosphonates are metabolic analogs that reduce macrophages. (3) Inhibition of tumor-promoting functions;^205,209–211^ Tim-3 blocking antibody is reported to regulate the activation of TAMs. By inhibiting angiogenesis, anti-VEGF, anti-VEGFR, and tyrosine kinase inhibitors could weaken the protumoral function of TAMs. TAMs contribute to an immunosuppressive microenvironment by expressing indoleamine-pyrrole 2,3-dioxygenase (IDO), heme oxygenase, arginase, TGFβ, IL-10, prostaglandins, and so on. Aspirin reduces the generation of prostaglandins. Blocking immune checkpoints (PD-L1, PD-L2, B7-H4, VISTA, B7-1, and B7-2) on macrophages could relieve the function of other immune cells. (4) Removal of the macrophage blockade;^207,212,213^ interactions between CD47 on tumors and SIRPα on macrophages help tumor cells evade macrophage phagocytosis. Antibodies against CD47 or SIRPα could remove the blockage. In addition, antibodies against MUC1 and EGFR inhibit SIRPα. (5) Induction of repolarization;^43,113,193,207,210,214–222^ M1 polarization of TAMs is associated with antitumor responses, while M2 polarization is associated with protumor activities. Several factors can induce M1 polarization, including IFNγ, CD40 agonists, inhibitors of PI3Kγ/mTOR/DICER, agonists of TLR4/7/8/9, methionine sulfoximine, histone deacetylase (HDAC) inhibitors, and antibodies against macrophage receptors with collagenous structures (MARCOs). In contrast, factors inhibiting M2 polarization, such as CSF1R inhibitors, corosolic acid, omeprazole, Gpr132 inhibitors, MEK/STAT3 inhibitors, fast-mimicking diets, and antibodies against IL-4, IL-4Rα, and IL-13, can also reduce the tumor burden. (6) Modification of effector cells.^177^ Chimeric antigen receptor macrophages (CAR-Ms) similar to CAR-T cells have been used to enhance tumoricidal functions. Targets, such as CD19, HER2, and mesothelin, have been explored

To date, great endeavors to boost T cell-directed anticancer immune responses have been made. As reported, the incidence of cancerogenesis is low in invertebrates with no T or B cells, indicating that innate immune cells are of great importance for preventing the initiation and development of cancer.^189–191^ In addition to their supporting role in all kinds of immunotherapies, macrophages may become a promising target in future cancer immunotherapy.^33,192^ Many targets and pharmacological agents related to macrophages in oncotherapy have been summarized in recent reviews.^128,193^ We updated the typical macrophages-targeting agents that have been registered for cancer-related clinical trials (excluding projects those are in the status of terminated, withdrawn, unknown, not yet recruiting) in Table 2. The potential and promising strategies targeting macrophages have been categorized into six types based on their objectives in Fig. 4. There are several advantages to target macrophages in cancer immunotherapy. Low infiltration is a major barrier for T-cell-based anticancer therapy, and macrophages account for ~30–50% of infiltrating immune cells in the tumor microenvironment. As mentioned above, circulating monocytes are a major source of infiltrating macrophages in tumors, and the accessibility of peripheral blood mononuclear cells makes it easy to operate if a macrophage-based therapy strategy is adopted in the clinic.

**Table 2:** Cancer-associated clinical trials (excluding projects those are in the status of terminated, withdrawn, unknown, not yet recruiting) using typical macrophage-targeting agents

Target	Agent	Organization	ClinicalTrials.gov Identifier	Tumors	Other interventions	Phase
CSF1	Lacnotuzumab (MCS110)	Novartis Oncology	NCT02435680	Advanced triple-negative breast cancer	Carboplatin, gemcitabine	II
			NCT01643850	Pigmented villonodular synovitis	None	II
			NCT03694977	Gastric cancer	PDR001	II
CCL2	Carlumab (CNTO 888)	Centocor Research & Development	NCT01204996	Solid tumors	Standard of care	I
			NCT00992186	Prostate cancer	None	II
SIRPα	TTI-622	Trillium Therapeutics	NCT03530683	Advanced relapsed or refractory lymphoma or myeloma	Rituximab, PD-1 inhibitor, proteasome-inhibitor regimen	I
	CC-95251	Celgene	NCT03783403	Advanced solid and hematologic cancer	None	I
	BI 765063 (OSE-172)	OSE Immunotherapeutics	NCT03990233	Advanced solid tumors	BI 754091	I
	FSI-189	Gilead Sciences	NCT04502706	Relapsed/refractory non-Hodgkin lymphoma	None	I
TIE2	CEP-11981 (ESK981)	Karmanos Cancer Institute	NCT04159896	Prostate cancer	Nivolumab	II
			NCT00875264	Advanced cancer	None	I
			NCT03456804	Prostate cancer	None	II
	Regorafenib (BAY 73-4506)	Bayer	NCT04170556	Hepatocellular carcinoma	Nivolumab	I/II
			NCT04476329	Hepatocellular carcinoma	None	II
	Arry-614	Array BioPharma	NCT01496495	Myelodysplastic syndromes	None	I
			NCT00916227	Myelodysplastic syndromes	None	I
Arginase	INCB001158 (CB1158)	Incyte	NCT03910530	Advanced solid tumors	None	I
			NCT02903914	Advanced/metastatic solid tumors	Pembrolizumab	I/II
			NCT03314935	Solid tumors	Oxaliplatin, leucovorin, 5-fluorouracil, gemcitabine, cisplatin, paclitaxel	I/II
			NCT03837509	Multiple myeloma	Daratumumab	I/II
HER2	CAR-macrophage	Carisma Therapeutics lnc.	NCT04660929	HER2 overexpressing solid tumors	None	I
GC vitamin D-binding protein	EF-022	Efranat	NCT02052492	Solid tumors	None	I
CD40	SEA-CD40	Seattle Genetics	NCT02376699	Solid tumors	Pembrolizumab	I
	APX005M	Apexigen	NCT03389802	Pediatric CNS	None	I
			NCT04130854	Locally advanced rectal adenocarcinoma	None	II
			NCT02482168	Non-small-cell lung cancer, melanoma, urothelial carcinoma, head and neck cancer	None	I
			NCT03165994	Esophageal cancer, gastroesophageal cancer	Radiation therapy, paclitaxel, carboplatin	II
			NCT03719430	Soft tissue sarcoma	Doxorubicin	II
			NCT03214250	Metastatic pancreatic Adenocarcinoma	Nivolumab, nab-paclitaxel, gemcitabine	I/II
			NCT04337931	Melanoma	None	II
			NCT02706353	Melanoma	Pembrolizumab	I/II
	CP-870,893	VLST Corporation	NCT01103635	Metastatic melanoma	Tremelimumab (anti- CTLA-4)	I
	Selicrelumab (R07009879)	Roche	NCT02760797	Advanced solid tumors	Anti-PD-L1	I
			NCT02665416	Advanced solid tumors	Bevacizumab or vanucizumab	I
			NCT02304393	Solid tumors	Atezolizumab	I
			NCT02588443	Pancreatic ductal adenocarcinoma	Gemcitabine, nab-paclitaxel	I
	CDX-1140	Celldex Therapeutics	NCT04491084	Non-small-cell lung cancer, lung cancer	CDX-301	I/II
			NCT04520711	Malignant epithelial neoplasms	TCR-T, pembrolizumab	I
			NCT04616248	Unresectable or metastatic breast cancer	Poly ICLC, radiation therapy, recombinant Flt3 ligand	I
			NCT04364230	Melanoma	6MHP, NeoAg-mBRAF, Poly ICLC	I/II
			NCT03329950	Advanced malignancies	CDX-301, pembrolizumab, chemotherapy	I
	Dacetuzumab (SGN-40)	Genentech, Inc. Seagen Inc.	NCT00525447	Multiple myeloma	Lenalidomide, dexamethasone	I
			NCT00079716	Multiple myeloma	None	I
			NCT00435916	Large B-cell diffuse lymphoma, non-Hodgkin lymphoma	None	II
			NCT00103779	Non-Hodgkin lymphoma	None	I
			NCT00655837	Large B-cell diffuse lymphoma, non-Hodgkin lymphoma	Rituximab, gemcitabine	I
			NCT00556699	Non-Hodgkin’s lymphoma	Rituximab	I
			NCT00664898	Multiple myeloma	Bortezomib	I
			NCT00283101	Lymphocytic, chronic leukemia	None	I/II
	Lucatumumab (HCD122)	Novartis Pharmaceuticals	NCT00670592	Non-Hodgkin’s lymphoma, Hodgkin’s lymphoma	None	I/II
			NCT01275209	Follicular lymphoma	None	I
			NCT00231166	Multiple myeloma	None	I
	2141 V-11	Rockefeller University	NCT04547777	Glioma	None	I
			NCT04059588	Solid tumor, skin cancer	D2C7-IT	I
	ADC-1013 (JNJ-64457107)	Janssen Research & Development, LLC	NCT02829099	Advanced solid neoplasms	None	I
	LVGN7409	Lyvgen Biopharma Holdings Limited	NCT04635995	Cancer	None	I
	Chi Lob 7/4	Cancer Research UK	NCT01561911	Neoplasms, lymphoma, non-Hodgkin, B cell	None	I
	NG-350A	PsiOxus Therapeutics	NCT03852511	Metastatic cancer, epithelial tumor	None	I
BTK	Ibrutinib (PCI-32765)	Pharmacyclics LLC	NCT02599324	Renal cell, urothelial, gastric, colon, pancreatic adenocarcinoma	None	Ib/II
			NCT01478581	Multiple myeloma	Dexamethasone	I
			NCT01752426	Leukemia	heavy water (2H_2_O)	I, II
			NCT01236391	Mantle cell lymphoma	None	II
			NCT01105247	B-cell chronic lymphocytic leukemia, small lymphocytic lymphoma	None	I, II
			NCT01614821	Waldenstrom’s macroglobulinemia	None	II
			NCT01292135	B-cell chronic lymphocytic leukemia, small lymphocytic lymphoma	None	I
			NCT01520519	Leukemia	Rituximab	II
			NCT01109069	B-cell lymphoma, chronic lymphocytic leukemia	None	II
			NCT01217749	Chronic lymphocytic leukemia	Ofatumumab	I, II
			NCT02403271	Non-small-cell lung cancer, breast cancer, pancreatic cancer	Durvalumab	I, II
			NCT01646021	Mantle cell lymphoma	Temsirolimus	III
			NCT01855750	Lymphoma	Rituximab, cyclophosphamide, doxorubicin, vincristine, prednisone	II
			NCT01980628	Marginal zone lymphoma, B-cell lymphoma	None	II
			NCT01589302	Prolymphocytic leukemia, small lymphocytic lymphoma, chronic lymphocytic leukemia	None	II
			NCT01325701	Diffuse large cell B lymphoma	None	II
			NCT01578707	Chronic lymphocytic leukemia, small lymphocytic lymphoma	Ofatumumab	III
			NCT01722487	Chronic lymphocytic leukemia, small lymphocytic lymphoma	Chlorambucil	III
			NCT02436668	Metastatic pancreatic adenocarcinoma	Gemcitabine, nab-paclitaxel	III
			NCT01980654	Follicular lymphoma, B-cell lymphoma, non-Hodgkin’s lymphoma	Rituximab	II
			NCT01973387	Chronic lymphocytic leukemia, small lymphocytic lymphoma	Rituximab	III
			NCT01611090	Chronic lymphocytic leukemia, small lymphocytic lymphoma	Bendamustine, hydrochloride, rituximab	III
			NCT02401048	Diffuse large B-cell lymphoma, follicular lymphoma	MEDI4736	I, II
			NCT02639910	Chronic lymphocytic leukemia, small lymphocytic lymphoma	Tafasitamab, idelalisi, venetoclax	II
			NCT02902965	Multiple myeloma	Bortezomib dexamethasone	II
			NCT01744691	Chronic lymphocytic leukemia with 17p deletion, small lymphocytic lymphoma with 17p deletion	None	II
			NCT02264574	Chronic lymphocytic leukemia, small-cell lymphoma	Obinutuzumab, chlorambucil	III
			NCT02514083	Chronic lymphocytic leukemia, small lymphocytic lymphoma	Fludarabine	II
	Acalabrutinib (ACP-196)	Acerta Pharma BV	NCT02112526	Activated B-cell diffuse large B-cell lymphoma	None	I
			NCT02180724	Waldenström macroglobulinemia	None	II
			NCT02213926	Mantle cell lymphoma	None	II
			NCT02211014	Multiple myeloma	None	I
	Zanubrutinib (BGB-3111)	BeiGene	NCT03206970	Mantle cell lymphoma	None	II
			NCT03206918	Chronic lymphocytic leukemia, small lymphocytic lymphoma	None	II
CSF1R	Pexidartinib (PLX-3397)	Plexxicon	NCT02371369	Tenosynovial giant cell tumor	None	III
			NCT02472275	Intermediate- or high-risk prostate cancer	None	I
			NCT02584647	Sarcoma, malignant peripheral nerve shealth tumors	Sirolimus	I
			NCT01596751	Metastatic breast cancer	Eribulin	Ib/II
			NCT02777710	Pancreatic or colorectal cancers	Durvalumab	I
			NCT02734433	Advanced solid tumors	None	I
			NCT03158103	Gastrointestinal stromal tumor	MEK162	I
	BLZ945	Novartis	NCT02829723	Advanced solid tumors	PDR001	I
	ARRY-382	Array Biopharma	NCT01316822	Metastatic cancer	None	I
			NCT02880371	Advanced solid tumors	Pembrolizumab	II
	Edicotinib (JNJ-40346527)	Johnson & Johnson	NCT03177460	Prostate cancer	None	I
	IMC-CS4(LY3022855)	Eli Lilly	NCT01346358	Advanced solid tumors	None	I
			NCT02265536	Advanced breast, prostate cancer	None	I
			NCT02718911	Solid tumor	Durvalumab, tremelimumab	I
			NCT03101254	Melanoma	Vemurafenib cobimetinib	I & II
			NCT03153410	Pancreatic ductal adenocarcinoma	Cyclophosphamide, pembrolizumab, GVAX	I
	Cabiralizumab (FPA008)	Five Prime Therapeutics	NCT02471716	Tenosynovial giant cell tumor	None	II
			NCT03927105	Peripheral T-cell lymphoma	Nivolumab	II
			NCT03502330	Melanoma, non-small-cell lung cancer, renal cell carcinoma	APX005M nivolumab	I
			NCT04331067	Triple-negative breast cancer	Nivolumab	Ib/II
			NCT03158272	Advanced malignancy	Nivolumab	I
			NCT02526017	Advanced solid tumors	Nivolumab	I
	Emactuzumab (RO5509554)	Hoffman La Roche	NCT02323191	Advanced solid tumors	Atezolizumab	I
			NCT02760797	Advanced solid tumors	RO7009789	I
			NCT01494688	Advanced solid tumors	Paclitaxel	I
			NCT03708224	Advanced head and neck squamous cell carcinoma	Atezolizumab	II
			NCT03193190	Pancreatic ductal adenocarcinoma	Additional therapies	I/II
	TPX-0022	Turning Point Therapeutics, Inc.	NCT03993873	Advanced solid tumor	None	I
	DCC-3014	Deciphera Pharmaceuticals LLC	NCT04242238	Sarcoma	Avelumab	I
			NCT03069469	Advanced malignant neoplasm	None	I & II
	Q702	Qurient Co., Ltd.	NCT04648254	Solid tumor	None	I
	SNDX-6532	Syndax	NCT03238027	Solid tumor	Durvalumab	I
			NCT04301778	Unresectable intrahepatic cholangiocarcinoma	Durvalumab	II
CD47	Magrolimab (Hu5F9-G4)	Gilead Sciences	NCT02216409	Solid tumor	None	I
			NCT03248479	Hematological Malignancies	Azacitidine	I
			NCT02678338	Acute myeloid leukemia, myelodysplastic syndrome	None	I
			NCT03527147	Non-Hodgkin’s lymphoma	AZD9150 acalabrutinib AZD6738 rituximab AZD5153	I
			NCT04599634	B-cell malignancies	Obinutuzumab venetoclax	I
			NCT02953782	Advanced solid malignancies and colorectal carcinoma	Cetuximab	I
			NCT03558139	Ovarian cancer	Avelumab	I
			NCT03248479	Hematological malignancies	Azacitidine	I
			NCT04541017	T-cell lymphoma	Mogamulizumab	I/II
			NCT03922477	Acute myeloid leukemia	Atezolizumab	I
			NCT04435691	Acute myeloid leukemia	Azacitidine, venetoclax	I/II
			NCT03869190	Urothelial carcinoma	Atezolizumab, enfortumab, vedotin, niraparib	I/II
			NCT02953509	Non-Hodgkin lymphoma	Rituximab, gemcitabine, oxaliplatin	I/II
			NCT04313881	Myelodysplastic syndromes	Azacitidine	III
	TTI-621	Trillium Therapeutics	NCT02890368	Solid tumors and mycosis fungoides	PD-1/PD-L1 inhibitor, pegylated interferon-α2a, radiation, talimogene laherparepvec	I
			NCT02663518	Small-cell lung cancer	None	I
	AO-176	Arch Oncology	NCT03834948	Solid tumor	Paclitaxel	I/II
			NCT04445701	Multiple myeloma	Bortezomib, dexamethasone	I/II
	IBI322	Innovent Biologics (Suzhou) Co., Ltd	NCT04328831	Advanced malignancies	None	I
			NCT04338659	Advanced malignancies	None	I
	ZL1201	Zai Lab (Shanghai) Co., Ltd.	NCT04257617	Locally advanced solid tumor	None	I
	CC-90002	Celgene	NCT02367196	Hematologic neoplasms	Rituximab	I
	HX009	Waterstone Hanxbio Pty Ltd	NCT04097769	Advanced solid tumor	None	I
	IBI188	Innovent Biologics (Suzhou) Co. Ltd.	NCT03717103	Advanced malignancies	Rituximab	I
			NCT03763149	Advanced malignancies	None	I
	SRF231	Surface Oncology	NCT03512340	Advanced solid cancers, hematologic cancers	None	I
	AK117	Akesobio Australia Pty Ltd	NCT04349969	Neoplasms malignant	None	I
	IMC-002	ImmuneOncia Therapeutics Inc.	NCT04306224	Solid tumor, lymphoma	None	I
CCR2	BMS-813160	Bristol-Myers Squibb	NCT03184870	Colorectal/pancreatic cancer	Chemotherapy or nivolumab	Ib/II
			NCT03496662	Pancreatic cancer	Nivolumab abraxane, gemcitabine	I/II
			NCT03767582	Pancreatic cancer	Radiation therapy, nivolumab, GVAX	I/II
			NCT04123379	Non-small-cell lung cancer, hepatocellular carcinoma	Nivolumab, BMS-986253	II
			NCT02996110	Advanced cancer	Nivolumab, ipilimumab, relatlimab, BMS-986205	II
	CCX872-B	ChemoCentryx	NCT03778879	Pancreatic cancer	Radiation therapy	II
	MLN1202	Millenium	NCT01015560	Bone metastases	None	II
	PF-04136309	Pfizer	NCT02732938	Metastatic pancreatic ductal adenocarcinoma	Nab-paclitaxel, Gemcitabine	II

Currently, it is generally believed that cancer cells originate from endogenous cells in humans. Even if numerous tumor-specific antigens have been identified, most specific antigens still exist in a few normal cells. In contrast, not all cancer cells express just one specific antigen because of tumor heterogeneity. Clearance of specific antigen-expressing cancer cells may only result in temporary and limited antitumor efficacy. Nevertheless, as a type of innate immune cell, macrophages can exert a tumor-suppressive function without targeting one specific antigen.^194,195^

Macrophages are a double-edged sword in the tumor microenvironment. As a prominent component of tumor stromal cells, macrophages can gather around blood vessels, induce angiogenesis, and promote tumor invasion. On the other hand, they could also phagocytose cancer cells and remodel the tumor microenvironment. Fortunately, the polarization of macrophages can be repolarized. The transformation from M2- to M1-phenotype macrophages is sufficient to cause a tumor-suppressive effect.^194–196^ Of note, the polarization of macrophages is independent of T cells, while M1 macrophages are able to induce Th1 immune responses, and M2 macrophages can trigger Th2 immune responses.^197^ This provides an opportunity to target macrophages in cancer immunotherapy. More importantly, the direction of macrophages to T or B cells does not rely on the existence of tumor-specific antigens. While IFN-γ from M1 macrophages is an incentive for Th1 responses, TGF-β and IL-10-derived M2 macrophages cause the generation of Treg cells.^32,113,197^

Trogocytosis is a process in which a tumor-derived antigen is transferred to Fcγ receptor-expressing lymphocytes with the help of certain antibodies. It has been demonstrated that tumor cells decrease the expression of specific antigens by delivering them to CAR-T cells or NK cells, leading to fratricide T cells or NK cells.^198,199^ Trogocytosis has also been discovered between tumor cells and macrophages and is partially responsible for tumor immune escape.^200,201^ However, Velmurugan et al. reported that persistent trogocytosis of macrophages eventually leads to the killing of antibody-opsonized tumor cells. They explained that these discrepancies might be caused by limited contact time between two types of cells and the lack of competing endogenous antibodies under physiological conditions.^202^ Moreover, macrophages are capable of presenting antigens. Proteins that have been passed to the plasma membrane by trogocytosis might be more likely to be processed and presented than cytosolic proteins.

In addition, as mentioned above, macrophages from different sources may exert different functions. This offers an opportunity for more accurately targeted immunotherapy. For example, CCR2^+^Ly6C^+^ inflammatory monocytes can be recruited to pulmonary metastasis sites by CCL2 secreted by tumor cells and then differentiate into Ly6C^−^ macrophages that promote metastasis.^101^ Selectively targeting this group of monocytes may reduce metastasis without damaging the homeostasis maintaining functions of residual macrophages.

Macrophages also have advantages in certain types of cancer. Approximately 20% of nonparenchymal cells in the liver are macrophages. Macrophages in different locations function differently. By stimulating adaptive immune responses, they exert tumoricidal or protumoral and, in general, protumoral functions.^203^ It has been summarized in a previous review that targeting pathogenic macrophages is a promising option for patients with liver disease.^204^ Moreover, ascites is a common pathological phenomenon in liver cancer that is often accompanied by a poor prognosis. Integrated single-cell RNA sequencing revealed that lymphocytes in ascites are similar to those in peripheral blood, while myeloid cells in ascites are more likely to originate from tumor-infiltrating myeloid cells. This notion was further confirmed by RNA velocity and phylogenetic trees of macrophages from various tissues. According to this study, intratumoral macrophage-based immunotherapy for hepatocellular carcinoma can not only resolve tumor burden in situ but also relieve ascites.

Thus, macrophages provide a force to be considered in tumor immunotherapy. Research on macrophages might open a new door for oncotherapy. To address various malignancies, more strategies based on or combined with macrophages need to be explored in the future.
